# The prevalence of obstructive sleep apnea-hypopnea syndrome in patients with multiple sclerosis: a systematic review and meta-analysis

**DOI:** 10.3389/fneur.2024.1444470

**Published:** 2024-12-17

**Authors:** Peipei Li, Jiaqi Liu, Jianying Yang, Jie He, Jiaqing Jiang

**Affiliations:** ^1^School of Clinical Medicine, Chengdu Medical College, Chengdu, Sichuan, China; ^2^Department of Otolaryngology-Head and Neck Surgery, The First Affiliated Hospital of Chengdu Medical College, Chengdu, Sichuan, China; ^3^Department of Pulmonary and Critical Care Medicine, The First Affiliated Hospital of Chengdu Medical College, Chengdu, Sichuan, China

**Keywords:** multiple sclerosis, obstructive sleep apnea-hypopnea syndrome, meta-analysis, incidence, sleep indexes

## Abstract

**Objective:**

Obstructive sleep apnea-hypopnea syndrome is the most common respiratory disorder in patients with multiple sclerosis. The purpose of this meta-analysis was to evaluate the prevalence of OSAHS in MS patients and to analyze their sleep monitoring indicators of patients.

**Methods:**

Online databases such as PubMed, EMBASE, Web of Science, CNKI, and WanFang were used to review the Chinese and English literature about OSAHS in MS patients in detail. Two researchers analyzed the Quality of included studies based on the Quality Assessment of Diagnostic Accuracy Studies. The prevalence and sleep monitoring data were analyzed using STATA 11.0 software. Based on the I^2^ values, pooled analyses were performed using either random (I^2^ > 50%) or fixed-effect models (I^2^ ≤ 50%).

**Results:**

Fourteen articles were selected for the final analysis. Our study shows that different diagnosis methods of OSAHS lead to different incidences. When the screening method was PSG, the incidence of OSAHS in MS patients was 36%; when the method was STOP-BANG, the incidence of OSAHS in MS patients was 26%; when the method was Berlin questionnaire, the incidence of OSAHS in MS patients was 30%. We performed subgroup analyses based on race, age, OSAHS severity, and BMI of patients with MS. The results suggested that the incidence of OSAHS was different in different subgroups of MS patients. In addition, we found that patients with MS generally had poorer sleep monitoring indicators.

**Conclusion:**

The current literature shows that the incidence of OSAHS is higher in MS patients. MS may affect the progression of OSAHS.

**Systematic Review Registration:**

https://www.crd.york.ac.uk/PROSPERO/display_record.php?RecordID=551500, CRD42024551500.

## 1 Introduction

The primary cause of non-traumatic neurological dysfunction in young people is multiple sclerosis (MS), an immune-mediated disease distinguished by demyelination and neurodegeneration within the central nervous system (CNS) (1). Sleep disorders are prevalent in up to 50% of individuals diagnosed with MS (2, 3). Several comorbidities were associated with increased hazard of death in MS patients, including diabetes, ischemic heart disease, depression, anxiety, and chronic lung disease (4). OSAHS is a common chronic pulmonary disease and also a prevalent sleep disorder (5), severely reducing quality of life and aggravating severe symptoms like exhaustion (6). Current evidence indicates that OSAHS disproportionately affects a substantial segment of the MS population (PwMS). In a specific clinic-based study, OSAHS was found in one-fifth of patients with MS, and more than half were identified as having a high risk for developing OSAHS (7). Despite these findings, research exploring the occurrence of OSAHS among individuals with MS remains limited.

Similar clinical manifestations that affect both patients with MS and OSAHS include fatigue, drowsiness, depression, and cognitive impairment. These clinical manifestations may have an impact on one another. Although some studies suggest that patients with MS have a higher prevalence and severity of OSAHS, the results reported in the current literature are contradictory (8). Additionally, the majority of accessible data originating from self-administered questionnaire studies, lack comprehensive analysis of polysomnography (PSG)-detected data. Consequently, the pathophysiological mechanisms of OSAHS in individuals with MS are poorly understood, and vice versa. Because OSAHS is linked to both local and systemic inflammation, there has been conjecture that this could worsen the impairment or progression of MS (9). Simultaneously, OSAHS and central sleep apnea may result from CNS damage due to MS, especially in areas of the brainstem that are vital for breathing (10).

The prevalence of OSAHS may vary among different subgroups of MS. Some recent studies have also reported sleep monitoring indicators in patients with MS. As a result, in order to assess the association between the incidence of OSAHS and MS objectively, a meta-analysis of all previous research is required. The pathophysiology of OSAHS in patients with MS as well as the relationship between the two conditions were examined and explored in this study. Additionally, this study performed a combined analysis of sleep monitoring-related indicators in patients with MS.

## 2 Materials and methods

### 2.1 Literature retrieval strategy

This meta-analysis has been registered with the Prospective Register of Systematic Reviews (PROSPERO). An extensive search was conducted across multiple databases, including PubMed, Web of Science, WanFang, CNKI, and EMBASE, to gather studies on the onset of MS in individuals diagnosed with OSAHS. The whole-time span from each database’s creation until May 1, 2024, was searched. Keywords and subject terms used were “multiple sclerosis” or “MS” and “obstructive sleep apnea-hypopnea syndrome” or “obstructive sleep apnea” or “OSA” or “OSAHS” or “OSAS”.

### 2.2 Study eligibility criteria

Two researchers independently assessed the literature’s content. Discrepancies in the research findings were settled by dialogue, negotiation, and advice from an impartial arbiter. Studies that satisfied the following requirements were added to the meta-analysis: (1) no language restriction on the literature; (2) published in a peer-reviewed journal; (3) included patients with MS and reported the prevalence of OSAHS; and (4) offered statistical findings (such as mean, standard deviation, and *p*-value) for evaluating the clinical traits, sleep tracking information, and demographic markers of patients with MS.

The diagnosis of OSAHS was established through polysomnography, the STOP-BANG questionnaire, or the Berlin questionnaire. Review articles, case reports, editorial letters, commentary, non-English papers, conference proceedings, theses, animal experiments, and research unrelated to the relevant literature were all disqualified throughout the selection process. The research with the highest sample size was included when two or more studies used the same database or sample. The OSAHS prevalence meta-analysis did not include studies that specifically reported matched-size groups since these studies might have influenced the overall prevalence findings. The quality of the studies was assessed using a modified version of the Quality Assessment of Diagnostic Accuracy Studies (QUADAS) instrument (11), which is more appropriate for prevalence studies and is frequently used. A study was deemed methodologically appropriate for inclusion in the meta-analysis if its score was more than 13 points.

### 2.3 Outcome measures

Primary outcome measure: Prevalence of OSAHS among patients with MS.

Secondary outcome measures:

1. Morbidity of OSAHS in patients with MS compared to those without MS.
2. Sleep-related indicators of the patients.

### 2.4 Literature selection

Each article’s title and abstract were carefully chosen by two writers on their own. Articles with potential relevance were thoroughly reviewed. Any differences of opinion among the writers were settled by consensus and, if required, by advice from a third author.

### 2.5 Data extraction and management

Two authors extracted the data on their own. The primary study or paper was included for studies with multiple publications, with additional information extracted from secondary sources as necessary. Authors were contacted to resolve data queries and disputes. In cases of duplicate publications, original authors were contacted to identify the main publication; if no response was received, the study with the highest participant count was selected. Each study’s initial author, publication year, research nation, participant characteristics, study design, and prevalence data were among the details that were extracted.

### 2.6 Statistical analyses

Pooled incidence and outcome measures were calculated through a random effects model employing an inverse variance Der Simonian Laird meta-analytical methodology (12). A proportion meta-analysis was performed to obtain pooled prevalence and incidences with 95% CIs. Inter-study differences in prevalence estimates were assessed with Higgins’ I^2^ statistics, where values exceeding 50% indicated moderate heterogeneity, as per established guidelines. Subgroup analyses and meta-regressions were performed to investigate the influence of variables on prevalence estimates. We conducted subgroup analyses for the primary outcome stratified by severity of OSAHS, BMI, age and race. The following elements were taken into account: To determine the mean and 95% confidence interval (CI) of sleep indicators for MS patients, single-arm meta-analyses were utilized. Higgins’ I^2^ statistic was once more used to measure inter-study heterogeneity; values more than 50% indicated significant heterogeneity. Funnel plots were used to assess publication bias in meta-analyses that contained 10 or more trials. We used Begg’s test and Egger’s regression test to look for signs of publication bias. Sensitivity analyses using the leave-one-out technique were carried out to determine which studies had a disproportionate impact on the final results. The impact of assessed factors on prevalence estimates and other outcomes was ascertained by meta-regression analysis. The Cochrane Collaboration’s RevMan 5.3^1^ and Stata version 11 for Windows (Stata Corp, College Station, Texas) were employed for all analyses.

## 3 Results

### 3.1 Literature retrieval and study selection

**Figure 1:**
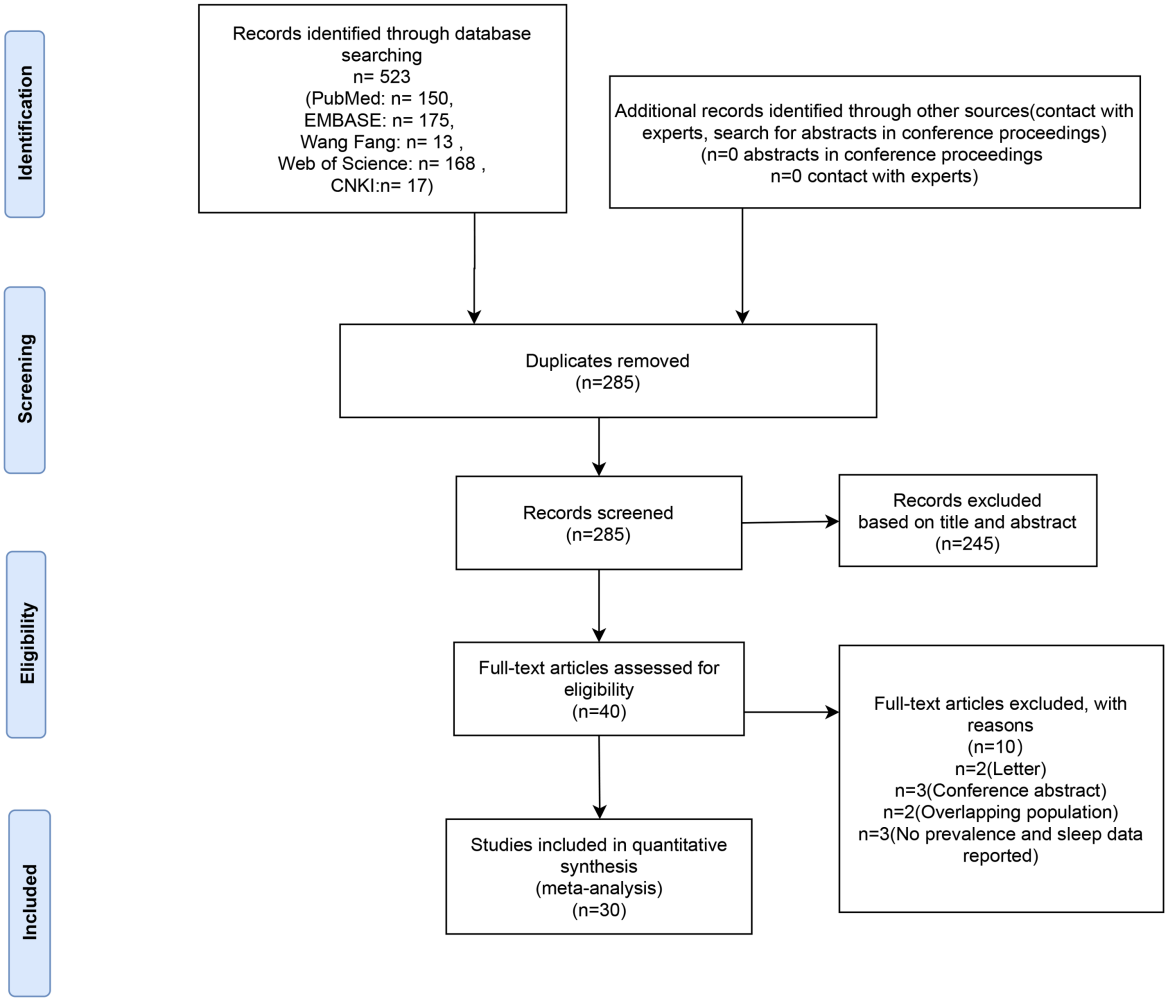
Flow diagram of literature screening.

Five hundred and twenty-three articles in all were retrieved after duplicates were eliminated. Out of all the articles, 40 were chosen for full-text evaluation based on their titles and abstracts. The meta-analysis included these 30 articles (7, 10, 13–40) as their quality ratings were higher than the QUADAS cut-off value (> 13). In Figure 1, the PRISMA flow chart is displayed.

Tables 1, 2 provide a summary of the clinical and sleep monitoring indicators of the MS population that were included in this meta-analysis. The investigations on the incidence of OSAHS among patients with MS originated from Europe (9 articles), North America (15 articles), Asia (4 articles), Africa (1 article), and Oceania (1 article), comprising a total of 6,447 patients with MS with OSAHS. Twenty--four articles, involving 28 studies, provided data on the incidence of OSAHS in patients with MS using methods such as PSG (*n* = 21), STOP-BANG (*n* = 9), and the Berlin Questionnaire (*n* = 4). Eleven articles reported apnea-hypopnea index (AHI) data for patients with MS. Fifteen articles included Epworth sleepiness scale data for patients with MS. Nine articles provided sleep efficiency data for patients with MS. Six articles offered sleep latency data for patients with MS. Four articles contained mean oxygen saturation data for patients with MS. Nine articles reported total sleep time data for patients with MS. Seven articles provided wake after sleep onset data for patients with MS. Six articles included STOP-BANG data for patients with MS.

**Table 1:** Characteristics of all eligible studies included in the meta-analysis.

Author	Year	Location	Study design	Mean age of cases (years)	Mean BMI of cases (kg/m^2^)	Men with MS (*n*)	*n*	*N*	Methods of OSAHS ascertainment
Terauchi T (OCST)	2024	Japan	CC	42.90 ± 9.30	23.70 ± 4.20	15	19	67	PSG
Queisi M (moderate/severe)	2024	USA	CSS	44	–	41	74	280	PSG
Queisi M (mild)				46.2	–	37	94	280	
Cousin C	2024	France	CS	56.10 ± 11.20	24.80 ± 4.40	56	95	125	PSG
Mazerolle M	2024	Canada	RCT	51.30 ± 9.40	28.90 ± 6.50	9	–	28	PSG
Valentine TR	2023	USA	CSS	48.28 ± 9.42	–	40	113	131	PSG
Braley TJ	2023	USA	CS	59.00 ± 4.30	27.30 ± 6.20	–	45	524	PSG
Yazdchi M	2022	Iran	CSS	34.29 ± 8.98	24.98 ± 3.78	31	2	103	STOP-BANG questionnaire
Sparasci D	2022	Switzerland	CSS	50.60 ± 8.20	25.40 ± 5.30	20	27	67	PSG
Singh M	2022	USA	CC	48.80 ± 10.90	31.60 ± 7.20	62	156	200	PSG
Hensen HA	2022	Australia	–	54.00 ± 8.00	29.00 ± 6.00	4	11	11	–
Mihalj M	2022	Croatia	CS	46.11 ± 9.76	26.51 ± 4.30	11	8	28	STOP-BANG questionnaire
Khadadah S	2022	Canada	RCT	49.60 ± 10.00	28.80 ± 6.70	12	–	34	PSG
Sunter G	2021	Turkey	CSS	39.92 ± 9.11	–	36	11	97	PSG
							24	97	STOP-BANG questionnaire
Whibley D	2021	UK	CSS	47.60 ± 10.00	–	19	–	49	PSG
Levit E	2020	USA	CSS	41.40 ± 1.90	32.70 ± 1.20	14	36	65	PSG
Shaygannejad V (SPMS)	2019	Iran	CS	43.52 ± 9.95	24.39 ± 5.53	–	8	50	STOP-BANG questionnaire Berlin questionnaire
Shaygannejad V (RRMS)	2019	Iran	CS	34.90 ± 8.36	24.92 ± 4.88	–	20	309	STOP-BANG questionnaire
							34	309	Berlin Questionnaire
White EK	2019	USA	CC	51.60 ± 12.13	–	159	167	531	PSG
Albertsdottir A	2019	Iceland	CSS	55.33 ± 15.68	–	–	56	234	STOP-BANG questionnaire
Abdel Salam OA	2019	Egypt	CS	31.12 ± 7.48	–	53	66	124	STOP-BANG questionnaire
							68	124	Berlin Questionnaire
Ma S	2017	China	CSS	40.22 ± 7.80	24.83 ± 4.60	96	85	231	PSG
Braley TJ	2016	USA	CSS	48.34 ± 10.12	–	17	33	38	PSG
González-Platas M	2016	Spain	–	43.45 ± 10.64	–	53	14	240	–
Veauthier C	2015	Germany	CSS	20–66	–	21	8	66	PSG
Sater RA	2015	USA	CS	45.66 ± 9.15	28.86 ± 6.44	8	12	32	PSG
Brass SD	2014	USA	CSS	54.70 ± 12.4	–	450	895	2,367	STOP-BANG questionnaire
							883	2,367	Berlin Questionnaire
Braley TJ	2014	USA	CSS	47.10 ± 12.10	22.60 ± 7.40	67	41	195	PSG
Kaminska M	2012	Canada	CSS	47.30 ± 10.40	24.00 ± 4.10	17	36	62	PSG
Dias RA	2012	USA	CS	45.78 ± 10.97	28.02 ± 6.49	29	43	103	STOP-BANG questionnaire
Braley TJ	2012	USA	CSS	47.60 ± 10.80	32.00 ± 5.20	16	27	42	PSG
Howard RS	1992	UK	CS	–	–	2	1	14	PSG

CC, case–control studies; CSS, cross-sectional studies; CS, cohort studies; RCT, randomized, controlled trial; PSG, polysomnography.

**Table 2:** Additional characteristics of case–control studies included in the meta-analysis.

Author	Year	MS group ED (*n*)/Total (*n*)	Control group ED (*n*)/Total (*n*)
Braley TJ	2023	45/524	4624/63342
Kaminska M	2012	36/62	15/32
Ma S	2017	85/231	41/265
Sparasci D	2022	24/67	17/67
Terauchi T	2024	19/67	8/31

### 3.2 Prevalence of OSAHS in participants diagnosed with MS

**Figure 2:**
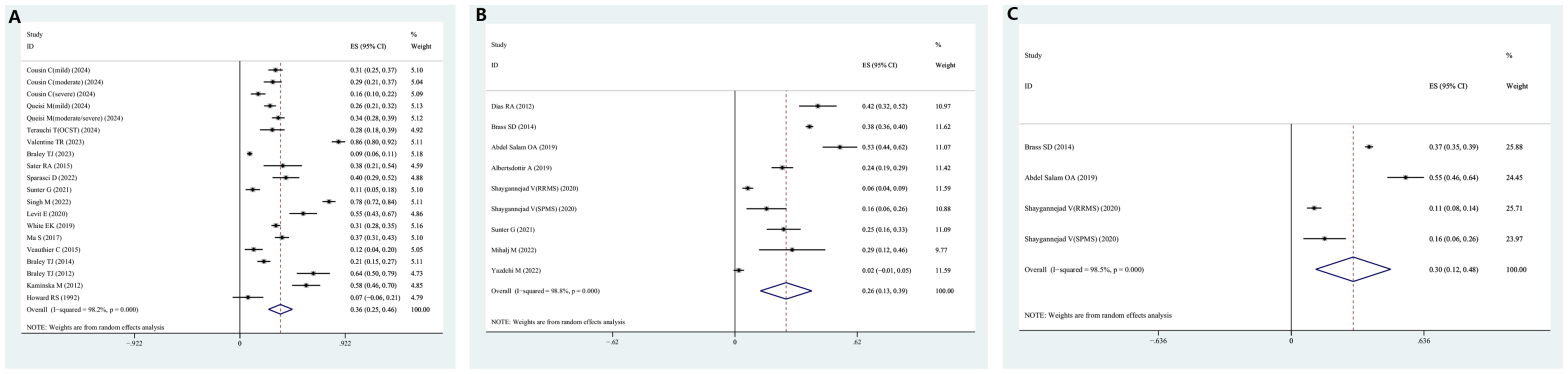
Forest plot of prevalence of OSAHS in patients with multiple sclerosis. **(A)** The OSAHS of diagnostic methods were PSG. **(B)** The OSAHS of diagnostic methods were STOP-BANG questionnaire; **(C)** The OSAHS of diagnostic methods were Berlin questionnaire.

Figures 2A–C illustrate the prevalence of OSAHS in participants diagnosed with MS. The overall prevalence of OSAHS across all studies was approximately 36% [95% confidence interval (CI): 25–46%] as determined by PSG (Figure 2A). The I^2^ value of 98.3% indicated significant heterogeneity among the studies. Supplementary Figure S1A presents the contour-enhanced funnel plot for the publication bias test, showing evidence of publication bias and symmetry. This was confirmed through Egger’s regression test (*p* = 0.083). Sensitivity analysis demonstrated that excluding any individual study did not affect the overall results of the combined analysis (Supplementary Figure S1B). The prevalence of OSAHS across all studies was about 26% [95% CI: 13–39%] according to the STOP-BANG questionnaire (Figure 2B). The prevalence of OSAHS was about 30% [95% CI: 12–48%] based on the Berlin questionnaire (Figure 2C).

#### 3.2.1 Subgroup analysis

Evidence indicates that factors such as the severity of OSAHS, body mass index (BMI), race, and age influence the morbidity of OSAHS. The subgroup analysis based on the severity of OSAHS revealed that among patients with MS, 28% had mild OSAHS, 29% had moderate OSAHS, 16% had severe OSAHS, and 34% had moderate to severe OSAHS. Most studies did not report the AHI values for patients with MS. The analysis based on BMI showed that patients diagnosed with both MS and obesity exhibited a higher morbidity of OSAHS compared to those with MS but without obesity (67% vs. 27%). As the average age of the included population was less than 65 years old, a subgroup analysis based on age could not be performed. Additionally, a subgroup analysis based on race indicated that Caucasian patients with MS had a similar morbidity of OSAHS compared to Asian patients with MS (35% vs. 34%) (Table 3).

**Table 3:** Subgroup analyses of morbidity of PSG diagnosis in different conditions.

Subgroup analysis of morbidity (*n*)	ES (95% CI)	*p* value	*I*^2^ (%)	*P*_h_
Overall (20)	0.36 (0.25,0.46)	<0.001	98.2%	<0.001
Severity				
Mild (2)	0.28 (0.24,0.33)	<0.001	86.2%	0.007
Moderate (1)	0.29 (0.21,0.37)	<0.001	–	–
Severe (1)	0.16 (0.10,0.22)	<0.001	–	–
Moderate/ Severe (1)	0.34 (0.28,0.39)	<0.001	–	–
Not mentioned (15)	0.38 (0.24,0.53)	<0.001	98.7%	<0.001
Mean BMI				
BMI < 30 (9)	0.27 (0.18,0.37)	<0.001	95.0%	<0.001
BMI ≥ 30 (3)	0.67 (0.52,0.82)	<0.001	83.9%	0.002
Not mentioned (8)	0.31 (0.14,0.48)	<0.001	98.3%	<0.001
Mean Age				
Age < 65 (19)	0.36 (0.25,0.47)	<0.001	98.3%	<0.001
Age ≥ 65 (0)	–	–	–	–
Not mentioned (1)	0.07 (−0.06,0.21)	0.301	–	–
Race				
Caucasian (17)	0.35 (0.22,0.48)	<0.001	98.5%	<0.001
Asian (2)	0.34 (0.26,0.42)	<0.001	42.7%	0.186
Mixed (1)	0.32 (0.28,0.36)	<0.001	–	–

ES, effect size; NA, not applicable; BMI, body mass index; P_h_, P _heterogeneity_. Severity: 1: mild; 2: moderate; 3: severe; 0: not mentioned; 4: Mixed. BMI: 1 ≥ 30; 2 < 30; 3: NA. Age: 1 ≥ 65; 2 < 65; 3: NA. Race: 1: Caucasian; 2: Asian; 3: Mixed.

### 3.3 Meta-regression analysis

Supplementary Table S1 identifies the factors correlated with morbidity estimates through meta-regression analysis. Although it was anticipated that meta-regression analysis would uncover sources of heterogeneity, none of the studied factors were found to be significantly correlated with morbidity estimates.

### 3.4 OSAHS in MS vs. OSAHS in non-MS

**Figure 3:**
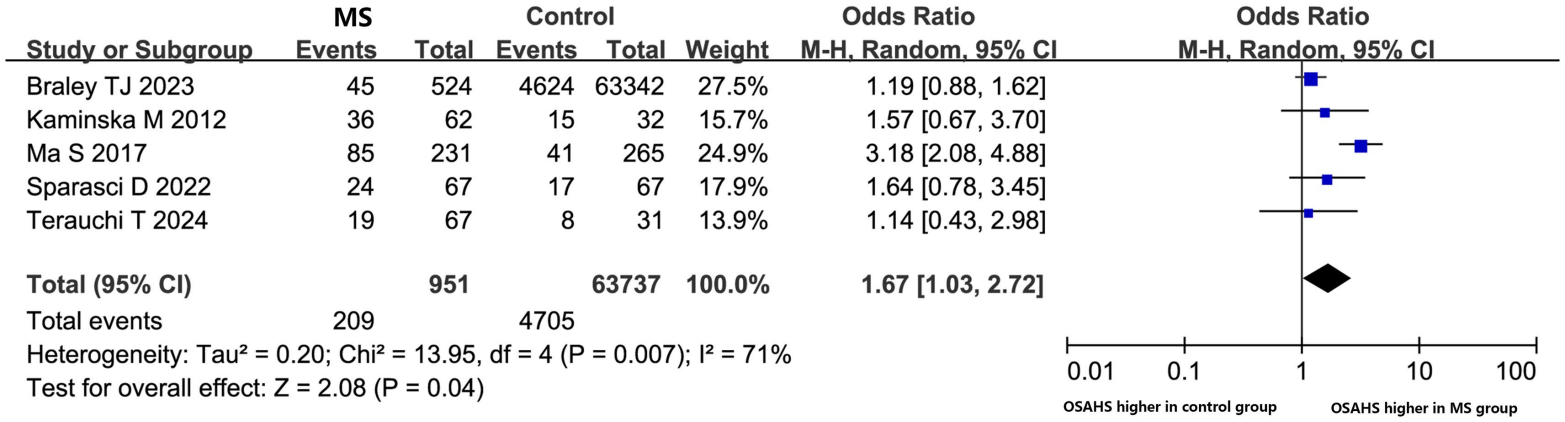
The prevalence of OSAHS in patients with multiple sclerosis compared to controls.

Nearly one billion individuals globally suffer from OSAHS, according to statistics (41). China has the highest number of patients with OSAHS globally, with over 176 million (23.6%) at high risk in the 30- to 69-year-old age range. In this research, five studies compared the morbidity of OSAHS in patients with MS to matched controls, most of which showed a higher prevalence of OSAHS among patients with MS (Figure 3). OSAHS has been demonstrated to be 1.67 times more common in patients with MS than in controls (OR: 1.67; 95% CI: 1.03–2.72; *p* = 0.04).

### 3.5 Sleep parameter indicators in patients with MS

#### 3.5.1 AHI

**Figure 4:**
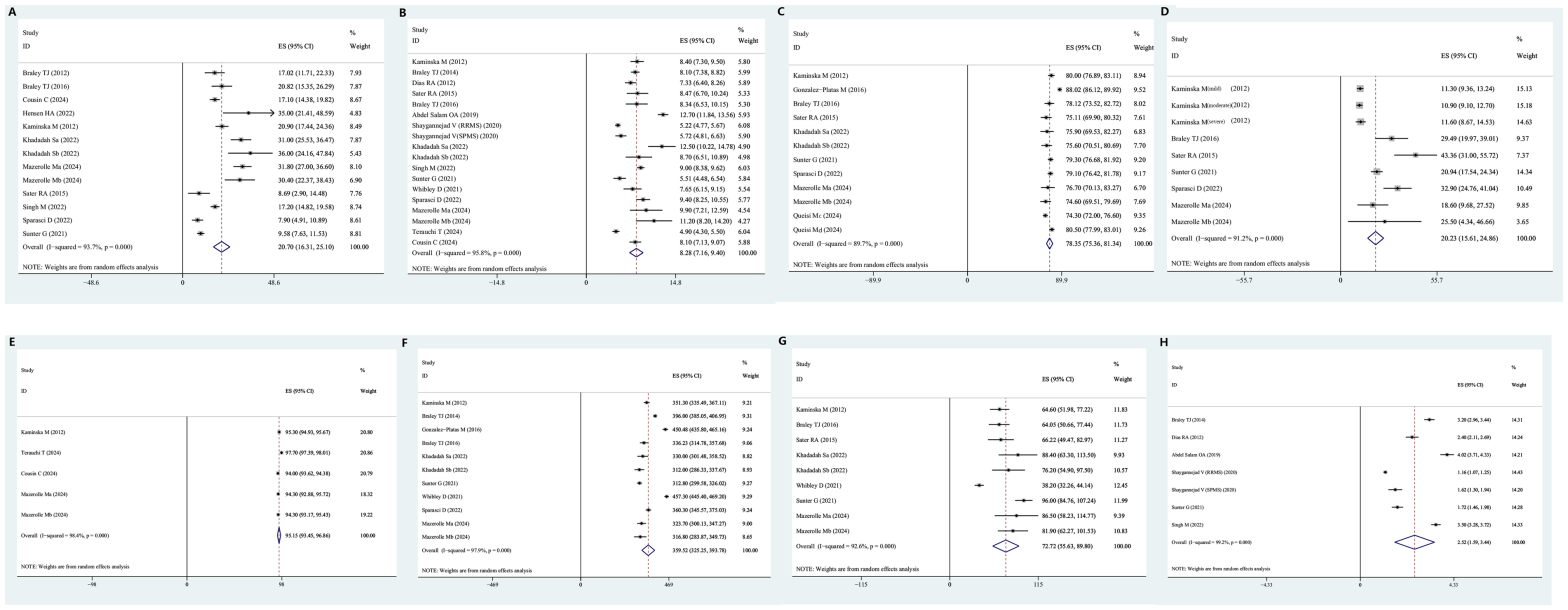
Forest plot and its 95%CI for eight key sleep monitoring parameters in OSAHS in patients with multiple sclerosis. **(A)** AHI, a: active CPAP group, b: Sham CPAP group. **(B)** Epworth sleepiness scale, a: active CPAP group, b: Sham CPAP group. **(C)** Sleep efficiency, a: active CPAP group, b: Sham CPAP group, c: MS duration >5 years, d: MS duration <5 years. **(D)** Sleep latency. **(E)** Average oxygen saturation, a: active CPAP group, b: Sham CPAP group. **(F)** Total sleep time, a: active CPAP group, b: Sham CPAP group. **(G)** Wake after sleep onset, a: active CPAP group, b: Sham CPAP group. **(H)** STOP-BANG score, RRMS, relapsing–remitting multiple sclerosis; SPMS, secondary progressive multiple sclerosis.

Thirteen studies provided the AHI for patients with MS. The combined analysis indicated that the average AHI in patients with MS was 20.70 (95% CI = 16.31–25.10) (Figure 4A).

#### 3.5.2 Epworth sleepiness scale

Eighteen studies provided Epworth sleepiness scale scores for patients with MS. The combined analysis revealed that the average Epworth Sleepiness Scale score among patients with MS was 8.28 (95% CI = 7.16–9.40) (Figure 4B).

#### 3.5.3 Sleep efficiency

Twelve studies provided data on sleep efficiency for patients with MS. The combined analysis indicated that the average sleep efficiency in patients with MS was 78.35 (95% CI = 75.36–81.34) (Figure 4C).

#### 3.5.4 Sleep latency

Nine studies provided data on sleep latency for patients with MS. The combined analysis showed that the average sleep latency in patients with MS was 20.25 min (95% CI = 15.61–24.86) (Figure 4D).

#### 3.5.5 Average oxygen saturation

Five studies provided data on average oxygen saturation for patients with MS. The combined analysis revealed that the average oxygen saturation in patients with MS was 95.15% (95% CI = 93.45–96.86%) (Figure 4E).

#### 3.5.6 Total sleep time

Eleven studies provided data on total sleep time for individuals with MS. The combined analysis showed that the total sleep time of individuals with MS was 359.52 min (95% CI = 325.25–393.78) (Figure 4F).

#### 3.5.7 Wake after sleep onset

Nine studies provided data on wake after sleep onset for patients with MS. The combined analysis indicated that wake after sleep onset in individuals with MS was 72.72 min (95% CI = 55.63–89.80) (Figure 4G).

#### 3.5.8 STOP-BANG score

Seven studies provided STOP-BANG questionnaire scores for patients with MS. The combined analysis revealed that the STOP-BANG score in patients with MS was 2.52 (95% CI = 1.59–3.44) (Figure 4H).

### 3.6 Descriptive analysis

Kaminska et al. (38) proved that MS patients had a higher risk of developing restless leg syndrome (RLS) than healthy controls (OR = 5.80, 95%CI = 1.51–38.2, *p* = 0.01), and, the wake after sleep onset time of MS patients was longer than healthy controls (64.6 ± 50.7 min vs. 40.1 ± 38.8 min, *p* = 0.01), the total arousal index of MS patients was higher than healthy controls (39.9 ± 15.9/h vs. 32.7 ± 13.4/h, *p* = 0.02). Ma et al. (31) conducted a cross-sectional self-report survey of 231 MS patients and 265 sex—and age-matched controls, which also indicated a higher incidence of restless leg syndrome in MS patients (21.6% vs. 5.3%). According to Braley et al. (10), patients with MS were prone to OSAHS and accompanying central apnea, and patients with MS had a higher AHI than healthy controls (17.02 ± 18.76 vs. 9.16 ± 8.84, *p* = 0.001).

## 4 Discussion

In recent decades, some epidemiological studies have identified a link between MS and the risk of OSAHS, though well-established information remains limited. The focus of this study was to evaluate the incidence of OSAHS in individuals with MS and determine if OSAHS is associated with MS at all. In the articles included in this analysis, the prevalence of OSAHS in patients with MS ranged from 7 to 86%. Analyses based on patient age, OSAHS diagnostic methods, BMI, and ethnicity across various studies continued to suggest a high incidence of OSAHS in patients with MS. The possible reasons are as follows: firstly, back pain and migraine are common and clinically significant comorbidities in MS that are often treated by painkillers (42, 43); medications (such as baclofen and carbamazepine) used to treat pain and spasms in MS may reduce pharyngeal muscle tone; secondly, the injury of specific areas of the central nervous system can be associated with sleep-related breathing disorders; Demyelinating lesions in the brainstem and spinal cord in MS may affect respiratory control and upper airway muscle activity, leading to OSAHS (44); thirdly, patients with MS may have neurological dysfunction, making them less active and more likely to become obese, while obesity and OSAHS form a vicious cycle; fourthly, because comorbidities of MS may require adequate therapeutic management, Patients with MS may experience polypharmacy, in which many drugs are administered simultaneously; Higher BMI and older age at onset were associated with major polypharmacy (45). Therefore, this study demonstrates that patients with MS have a higher risk of developing OSAHS compared to patients with non-MS (37). Studies have shown that MS-related lesions in the CNS contribute to the development of OSAHS. However, the prevalence of OSAHS is also high in the general population (41), with increasing age, obesity, and male gender being pivotal risk factors (46). These risk factors may also influence OSAHS in patients with MS, although their impact on patients with MS appears to differ from that on the non-MS population. Notably, although the prevalence of OSAHS in the general population (47) exhibits gender differences, with a prevalence of 7% in adult males and 5% in adult females, and up to 33% in some populations (48), OSAHS is often underdiagnosed in clinical practice (49). This suggests that although the incidence of OSAHS is high, it may not be a characteristic of MS.

Multiple sclerosis patients with comorbid OSAHS may present distinctive clinical phenotypes and neuroimaging alterations. Levit et al. (26) conducted a retrospective analysis of 65 multiple sclerosis patients who underwent either in-laboratory PSG or home sleep apnea testing to assess fatigue, followed by an MRI within 1 year of the sleep study. The MRI evaluation considered both the quantity and distribution of lesions in the brain and brainstem, as well as the standardized third ventricular width, which serves as an indicator of brain atrophy. Patients diagnosed with OSAHS were found to be slightly but significantly older, exhibited higher BMI, had a greater incidence of hypertension, marginally higher EDSS scores, and increased TVW (indicative of greater atrophy) compared to those without OSAHS. Notably, univariate analysis showed that MS patients with OSAHS had significantly more pontine lesions, although there were no significant differences in the midbrain or medullary lesions compared to non-OSAHS patients. In adjusted multivariate analyses, the apnea-hypopnea index was strongly associated with midbrain and pontine lesions, but not with medullary lesions, cerebral lesions, or TVW. Abdel Salam et al. (30) also compared MS patients diagnosed with high risk of OSAHS and those without OSAHS, and found significant differences between the two groups regarding EDSS and the presence of brainstem lesions. Multiple sclerosis patients with high OSAHS risk were more commonly suffering from fatigue and excessive daytime sleepiness than those without. Kaminska et al. (38) evaluated the relationship of OSAHS to fatigue and sleepiness in MS patients and found that OSAHS was frequent in MS and was associated with fatigue but not sleepiness, independent of MS-related disability and other covariates. Unfortunately, there was insufficient extractable data mentioned above, preventing us from conducting a meta-analysis to compare the relationship between EDSS, brain volume, and mental fatigue between MS patients with OSAHS and MS patients with non-OSAHS.

In the present study, it was also found that the incidence of OSAHS was higher (67%) in obese patients (BMI ≥ 30 kg/m^2^). BMI is a reliable indicator for measuring body fat and fat mass. A BMI value higher than 25 indicates that being overweight leads to upper airway narrowing and collapse, which is an important factor contributing to the occurrence of OSAHS (50, 51). Similar to the general population, weight gain appears to be a risk factor for OSAHS in patients with MS. This relationship is also observed in patients with MS. In a study by Hensen et al. (25), the incidence of OSAHS was lower in reports with lower BMI (BMI = 23–28 kg/m^2^) and higher in reports with higher BMI (26–32 kg/m^2^) in the MS prevalence study. Analyzing the reasons, In the present meta-analysis, the incidence of OSAHS in Asian and Caucasian MS populations does not differ markedly, which is related to the gradual increase in obesity rates in Asian populations in recent years (52).

Fatigue is prevalent among patients with MS and can lead to severe disability (53). Multiple sclerosis is a chronic inflammatory disease characterized by demyelinated lesions in both the grey matter (GM) and white matter (WM) of the central nervous system (54). Fatigue is generally thought to result from distributed lesions of the WM resulting in global impairment of brain function (55). According to diffusion-weighted imaging studies, WM changes are associated with fatigue, particularly in the anterior internal capsule and anterior thalamus (56–58). GM damage can also lead to fatigue. First, GM damage may disrupt large-scale networks in the brain responsible for regulating motor and cognitive processes (59); second, deep GM damage may affect structures involved in vigilance, arousal, and motivation, such as the hypothalamus, which can influence wakefulness and fatigue (60, 61); third, GM damage may cause persistent dysfunction in bodily systems by disrupting the regulation of the endocrine and autonomic nervous systems, further contributing to fatigue (62, 63). Additionally, peripheral immune and inflammatory processes in MS may also play an important role in fatigue (64, 65). Moreover, MS-related fatigue is associated with maladaptive network recruitment during task performance and metacognitive interpretations of brain states that suggest “helplessness” (55). MS is characterized by cognitive dysfunction, affecting approximately 40 to 60% of patients at some point during the disease (66). Despite the relative sparing of other neurological deficits, Staff et al. (67) emphasize the importance of considering MS as a cause of acute or progressive severe cognitive impairment. Compared to the general population, patients with MS have a greater frequency of sleep disorders (68, 69). These sleep disorders include OSAHS, insomnia, RLS, and periodic limb movement disorder. OSAHS can also cause fatigue and cognitive dysfunction (70). Quite notably, the COVID-19 pandemic has greatly impacted MS patient care and treatment across many settings. COVID-19 infection was unpredictable and difficult to avoid in patients with MS. Therefore, in a significant percentage of MS patients, post-COVID symptoms such as daytime sleepiness, fatigue and memory and concentration problems may be due to reduced sleep efficiency and sleep apnea (71). Braley et al. (33) assessed 38 MS patients from an outside patient clinic that asked about sleep or cognition during routine visits. They demonstrated an association between several components of neuropsychological function (attention and working memory) and oxygen desaturation index, minimum oxygen saturation, and respiratory disturbance index. OSAHS and MS influence and promote each other, forming a vicious circle. Systemic and neuroinflammation associated with MS may lead to sleep abnormalities and disturbances, while sleep fragmentation may worsen MS neuropathology or symptoms. Research suggests that OSAHS is associated with endogenous melatonin dysregulation, lesions in the accessory motor area, and a higher spinal cord lesion burden (15). By promoting systemic inflammation, OSAHS can compromise the integrity of white matter. Poor sleep quality may lead to oxidative stress, exerting toxic effects on oligodendrocytes and the myelin sheath’s gradual destruction, which may worsen MS symptoms. Additionally, poor sleep quality can also affect the course of relapsing–remitting MS (72). In a cohort of individuals with MS assessed using the Pittsburgh Sleep Quality Index, poor sleepers were significantly associated with an increased number and duration of MS relapses and an increase in MS active days (73).

Actively treating respiratory comorbidities in MS patients is highly beneficial for them. SARS-COV-2 humoral immune responses are still maintained in MS patients even when treated with anti-CD20 agents but to a lesser extent. Since immune memory involves B lymphocytes as well as CD4+ and CD8+ T cells, the attainment of protection against reinfection is plausible in these patients (74). Some studies suggest that continuous positive airway pressure (CPAP) therapy enhances executive function, speech development delay, visual memory, attention, alertness, and overall cognitive function in patients with OSAHS (75). The Paced Auditory Serial Addition Test (PASAT) and other apnea indicators have shown improvements in scores with CPAP treatment (76). Patients with OSAHS may exhibit white matter lesions on magnetic resonance imaging (MRI) and cognitive decline on PASAT, both of which can be improved with CPAP treatment (77). Continuous use of CPAP not only alleviates clinical symptoms in patients with MS but also aids in the recovery of brain structure. These findings indicate that cognitive decline due to OSAHS in MS and OSAHS patients can be mitigated with CPAP. Actively screening for OSAHS in patients with MS and providing early intervention for OSAHS is favorable for patients with MS.

Importantly, an increasing amount of evidence indicates that the development and progression of disability in MS may be influenced by hypoxia and/or inadequate arterial perfusion, in addition to related cardiovascular risk factors such as diabetes, smoking, and hypertension (78). Direct ischemia or downstream inflammatory pathways associated with OSAHS (79) could potentially influence the course of MS. The longitudinal effects of OSAHS on MS are still poorly understood, despite these new findings and the obvious connections between OSAHS and cardiovascular risk. A possible connection between sleep pathology and visual memory impairment in MS has been suggested by Valentine (17), who examined the relationship between cognitive performance in PwMS and sleep disorders based on PSG. She discovered significant associations between apnea severity and N2 sleep with immediate and delayed visual memory phenotypes.

In the general population, the Berlin questionnaire (80) and the STOP-BANG (81) are both commonly employed to determine the risk of OSAHS. These questionnaires assess key OSAHS symptoms, such as snoring and breathing pauses, and include anthropometric characteristics associated with OSAHS risk, such as male gender and hypertension, which could be especially pertinent in the MS community. Additionally, patients who experience fatigue may misunderstand queries concerning drowsiness. Data on the diagnostic accuracy of questionnaire-based diagnoses are limited, particularly in MS, and many studies on OSAHS prevalence have not validated the questionnaires used against PSG (9). For instance, in a cohort analysis of 62 patients with MS (38), where 48 showed OSAHS on PSG, the sensitivity of the Berlin questionnaire was only 46%, although its specificity for predicting OSAHS was 86%. Singh et al. (7) suggested that STOP-BANG may be an effective screening tool for OSAHS, especially in patients with severe OSAHS. Although questionnaire-based study results should be interpreted cautiously, most studies suggest that the percentage of patients with MS developing OSAHS ranges from 38 to 47% (72). The subgroup analysis based on different OSAHS diagnostic methods in this meta-analysis also shows that the prevalence of OSAHS in MS varies. Moreover, the average STOP-BANG questionnaire score for individuals with MS, according to this study, is 2.52, which is near the 3-point cutoff, suggesting a higher frequency of OSAHS in individuals with MS. Patients with MS who experience extreme exhaustion and low subjective sleep quality should also be evaluated for the potential of simultaneous OSAHS in addition to snoring and breathing pauses. This study also suggests that patients with MS have higher Epworth sleepiness scale scores and lower sleep efficiency.

In a recent study, increasing age was identified as a risk factor for MS. Most studies report a higher prevalence of sleep disorders when the mean age of MS subjects is 45 years or older, while studies with a mean age below 43 years report a lower prevalence of sleep disorders (25). This study used a cut-off value of 65 years to analyze the prevalence of OSAHS in elderly and non-elderly patients with MS. Unfortunately, most of the included studies focused on non-elderly patients with MS. The research that is now available makes it unclear whether age influences the severity of OSAHS along the course of the disease or whether it encourages the development of OSAHS in MS through processes similar to those in the general population.

Current research indicates that neuropathological alterations in the brainstem, associated with MS, play a marked role in the onset or exacerbation of OSAHS. This study reveals that in addition to average oxygen saturation, eight key sleep monitoring parameters related to MS are inferior compared to healthy individuals (10, 25, 26, 82). Recent investigations have explored the association between OSAHS and CNS lesions in patients with MS. The AHI was significantly elevated in MS patients with brainstem lesions compared to the control group, suggesting that MS-related brainstem lesions contribute to the development of OSAHS in this population. The distribution of brainstem lesions in MS patients (10, 26, 82) with OSAHS implies that MS may also influence altered arousal responsiveness to respiratory stimuli and ventilatory loop gain (83–85). The external lateral parabrachial nucleus, situated in the dorsolateral pons, is essential for mediating arousal responsiveness to CO_2_, hypoxia, and other respiratory stimuli (26, 84). Consequently, MS lesions affecting this region (26) could modify arousal responses. Furthermore, MS involvement of other midbrain and pontine nuclei, which regulate sleep and wakefulness (10, 26), could impact sleep–wake stability and arousal responsiveness (84). This may explain the prolonged wake after sleep onset times, higher total arousal index, and poorer sleep monitoring indicators observed in most patients with MS.

The heterogeneity of results in meta-analyses is often associated with various factors, including the quality of the included studies, population characteristics, diagnostic criteria for the disease, and other elements. In the present study, high heterogeneity was observed in most of the combined analyses. To investigate the potential origins of heterogeneity, subgroup analyses and meta-regression were conducted on the meta-analysis with the largest sample size concerning the prevalence of OSAHS. The meta-regression analysis indicated that race, disease severity, body weight, and age were not the sources of heterogeneity. Nonetheless, subgroup analyses based on these factors were performed, but no apparent sources of heterogeneity were identified. In the general population, the prevalence of OSAHS is twice as high in males compared to females (86). In contrast, MS is more prevalent in females. Consequently, the male-to-female ratio is a crucial factor to consider when interpreting the prevalence of OSAHS in MS patients. However, most studies did not provide gender-specific prevalence data for OSAHS. Moreover, heterogeneity may stem from variations in recruitment criteria, participant characteristics, the use of complete laboratory PSG versus home sleep testing devices, different airflow sensor technologies, and scoring criteria for sleep record evaluation. Additional research is necessary to more precisely determine the prevalence of OSAHS in diverse MS populations. Furthermore, unknown confounding factors may contribute to the increased heterogeneity of the meta-analysis, thereby reducing its reliability.

There are several advantages to this meta-analysis. First, it implies that there is a greater likelihood of concomitant OSAHS in those with MS. When assessing patients with MS, clinicians should also consider their sleep status, as treating OSAHS may help control MS symptoms. Patients with MS can benefit from OSAHS evaluation. This is the most extensive meta-analysis of pertinent literature, and the findings drawn from subgroup analyses are stronger. Second, sleep-related test indicators in patients with MS were analyzed, giving future researchers an intuitive understanding of sleep status in MS. Third, all included articles were of medium to high quality, enhancing the reliability of this meta-analysis.

However, this study has some potential limitations. First, the systematic review analyzed sleep monitoring index data of patients with MS, but all included studies were single-arm trials with a high risk of selection bias. Trial Sequential Analysis (TSA) is a method that incorporates the concepts of early termination and interim analysis from group sequential designs into meta-analysis. TSA assesses whether the sample size of the studies included in the current meta-analysis is sufficient through the estimated required information size (RIS). Although TSA plays an important role in meta-analysis, it also has certain limitations. For example, the TSA software can only perform analyses on binary and continuous data. However, most of the studies included in this research are single-arm studies, which means that in these specific study designs, TSA may not be directly applicable. Thus, we were unable to perform sample size estimates on subgroup populations. Second, pivotal confounding factors were not considered, which may lead to publication bias. Additionally, most study populations were Caucasian, with a relatively small sample size of Asian populations, making it hard to make firm conclusions on the independent influence of MS on OSAHS sleep monitoring results. Furthermore, due to the lack of specific AHI values in most studies, it is not possible to interpret the harm caused by various severities of OSAHS in patients with MS. The presence of vascular comorbidities and age-related disorders could play a role in OSAHS. However, we were not able to perform subgroup analyses by vascular comorbidities and age-related disorders, owing to restrictions with the currently available data. According to the variable progression of MS, four major clinical types are classified including relapsing remitting MS (RRMS), progressive MS (PMS), secondary progressive MS (SPMS), and progressive MS (PPMS). Unfortunately, Due to the lack of data on MS type, we could not conduct a subgroup analysis based on the clinical types of MS. Finally, the lack of effective longitudinal cohort studies in this research prevents us from inferring the causal relationship between OSAHS and MS.

## 5 Conclusion

In conclusion, this meta-analysis indicates that the proportion of individuals with MS with comorbid OSAHS is relatively high, and sleep monitoring indicators are worse in individuals with MS. Therefore, it is necessary to screen patients with MS for OSAHS.

## Data Availability

The original contributions presented in the study are included in the article/Supplementary material, further inquiries can be directed to the corresponding author.

## Footnotes

^1^ http://tech.cochrane.org/revman

## Supplementary material

SUPPLEMENTARY FIGURE S1 Sensitivity analysis and funnel plots for prevalence of OSAHS in patients with multiple sclerosis. **(A)** Sensitivity analysis; **(B)** Funnel plots of Begg’s test.
