# Supplementary figures and images for: The prevalence of obstructive sleep apnea-hypopnea syndrome in patients with multiple sclerosis: a systematic review and meta-analysis
Source: Front Neurol. 2024 Dec 17;15:1444470. doi: 10.3389/fneur.2024.1444470 (PMC11685047; doi:10.3389/fneur.2024.1444470)

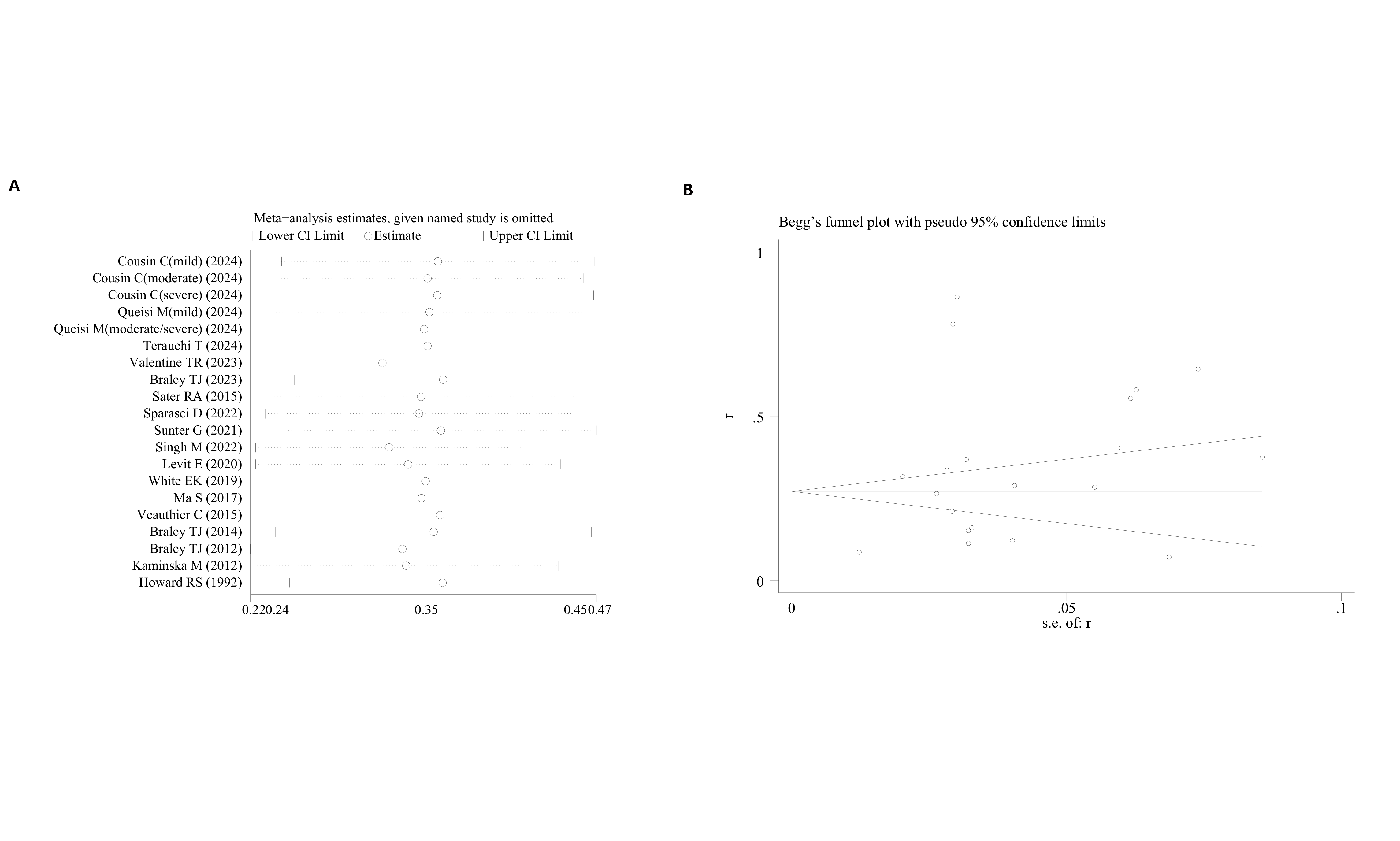

Supplement: SUPPLEMENTARY FIGURE S1 — Sensitivity analysis and funnel plots for prevalence of OSAHS in patients with multiple sclerosis. (A) Sensitivity analysis; (B) Funnel plots of Begg’s test. [file Image_1.tif]
