# Supplementary material for: The prevalence of obstructive sleep apnea-hypopnea syndrome in patients with multiple sclerosis: a systematic review and meta-analysis
Source: Front Neurol. 2024 Dec 17;15:1444470. doi: 10.3389/fneur.2024.1444470 (PMC11685047; doi:10.3389/fneur.2024.1444470)
Supplement: Supplementary file 2 [file Supplementary_file_1.docx]

**Table S1 Factors associated with OSAHS prevalence estimates identified by meta-regression analysis.**

| **Factor** | **OR (95% CI)** | **p-value** | **Explained variation (%)** |
| --- | --- | --- | --- |
| BMI |  |  | 2.4 |
| < 30 | 1 (reference) |  |  |
| > 30 | 0.64 (0.38 to 4.23) | 0.612 |  |
| Not reported | 1.01 (0.27 to 6.58) | 0.752 |  |
| Ethnicity |  |  | 1.2 |
| Caucasian | 1 (reference) |  |  |
| Asian | 0.92 (0.23 to 4.17) | 0.367 |  |
| Mixed | 1.25 (0.26 to 5.58) | 0.536 |  |
| Diagnostic criteria |  |  | 4.0 |
| PSG | 1 (reference) |  |  |
| STOP-BANG | 0.75 (0.16 to 4.56) | 0.726 |  |
| Berlin Questionnaire | 1.14 (0.20 to 5.41) | 0.833 |  |
| Severity |  |  | 0.0 |
| Mild | 1 (reference) |  | 0.0 |
| Moderate | 0.90 (0.13 to 2.27) | 0.323 |  |
| Severe | 1.04 (0.15 to 3.78) | 0.419 |  |
| Not reported | 0.88 (0.21 to 568) | 0.538 |  |
